# Expression of Melatonin Receptor 1 in Rat Mesenteric Artery and Perivascular Adipose Tissue and Vasoactive Action of Melatonin

**DOI:** 10.1007/s10571-020-00928-w

**Published:** 2020-07-30

**Authors:** Lubos Molcan, Andreas Maier, Anna Zemančíková, Katharina Gelles, Jozef Török, Michal Zeman, Isabella Ellinger

**Affiliations:** 1 Department of Animal Physiology and Ethology, Faculty of Natural Sciences, Comenius University Bratislava, Bratislava, Slovakia; 2 Institute for Pathophysiology and Allergy Research, Center for Pathophysiology, Infectiology and Immunology, Medical University Vienna, Waehringer Guertel 18–20, 1090 Vienna, Austria; 3 Institute of Normal and Pathological Physiology, Centre of Experimental Medicine, Slovak Academy of Sciences, Bratislava, Slovakia

**Keywords:** Rat melatonin receptor MT_1_, Melatonin, Mesenteric artery (MA), Perivascular fat tissue (PVAT), Neurogenic contraction

## Abstract

Melatonin is released by the pineal gland and can modulate cardiovascular system function via the G protein-coupled melatonin receptors MT_1_ and MT_2_. Most vessels are surrounded by perivascular adipose tissue (PVAT), which affects their contractility. The aim of our study was to evaluate mRNA and protein expression of MT_1_ and MT_2_ in the mesenteric artery (MA) and associated PVAT of male rats by RT-PCR and Western blot. Receptor localization was further studied by immunofluorescence microscopy. Effects of melatonin on neurogenic contractions were explored in isolated superior MA ex vivo by measurement of isometric contractile tension. MT_1_, but not MT_2_, was present in MA, and MT_1_ was localized mainly in vascular smooth muscle. Moreover, we proved the presence of MT_1_, but not MT_2_ receptors, in MA-associated PVAT. In isolated superior MA with intact PVAT, neuro-adrenergic contractile responses were significantly smaller when compared to arteries with removed PVAT. Pre-treatment with melatonin of PVAT-stripped arterial rings enhanced neurogenic contractions, while the potentiating effect of melatonin was not detected in preparations with preserved PVAT. We hypothesize that melatonin can stimulate the release of PVAT-derived relaxing factor(s) via MT_1_, which can override the direct pro-contractile effect of melatonin on vascular smooth muscle. Our results suggest that melatonin is involved in the control of vascular tone in a complex way, which is vessel specific and can reflect a sum of action on different layers of the vessel wall and surrounding PVAT.

## Introduction

Melatonin (5-methoxy-*N*-acetyltryptamine) is released by the pineal gland during night-time and impacts on many aspects of human physiology (Singh and Jadhav 2014), including the cardiovascular system (Sun et al. 2016; Tengattini et al. 2008). A meta-analysis of randomized controlled trials revealed that controlled release of melatonin significantly decreased systolic and diastolic blood pressure (BP) (Grossman et al. 2011). Night-time supplementation with melatonin reduced nocturnal BP in untreated hypertensive men (Scheer et al. 2004), non-dipping women (Cagnacci et al. 2005), or patients with nocturnal hypertension (Grossman et al. 2006). Importantly, no serious adverse events due to melatonin treatment were reported in all studies included in the meta-analysis (Grossman et al. 2011). Moreover, non-dipping hypertensive patients exhibit a reduced nocturnal melatonin secretion when compared to dipping hypertensive patients (Jonas et al. 2003; Zeman et al. 2005). These results suggest that melatonin can improve nocturnal BP control and may be especially useful for high-risk patients with nocturnal hypertension (Simko et al. 2016).

The mechanisms of melatonin-dependent modulation of BP remain poorly understood. Two mechanisms of action have been proposed: first, melatonin, being a molecule with an anti-oxidative capacity, can enhance NO synthase activity, reduce oxidative stress, and thus affect BP (Pechanova et al. 2007; Tengattini et al. 2008). Second, melatonin may impact on local BP regulation in peripheral vessels via the Class A (Rhodopsin-like) G protein-coupled melatonin receptors MT_1_ and MT_2_. Expression of these receptors has been shown in the cardiovascular systems of rats (Benova et al. 2009; Chucharoen et al. 2003; Masana et al. 2002; Sallinen et al. 2005; Schepelmann et al. 2011; Viswanathan et al. 1997), cows (Chucharoen et al. 2007), pigs (Tunstall et al. 2011), and humans (Ekmekcioglu et al. 2001, 2003). The receptors were found in different vessels such as coronary arteries (Ekmekcioglu et al. 2001, 2003; Tunstall et al. 2011), cerebral arteries (Chucharoen et al. 2003; Savaskan et al. 2001; Viswanathan et al. 1997), thoracic aorta (Benova et al. 2009; Schepelmann et al. 2011), and caudal arteries (Masana et al. 2002). Although co-expression of both receptors in some vessels has been observed (Masana et al. 2002; Schepelmann et al. 2011), it appears that other vessels express only either MT_1_ (Schepelmann et al. 2011) or MT_2_ (Tunstall et al. 2011).

The net effect of melatonin on blood vessels (relaxation, contraction) may, therefore, depend on both the type of expressed receptor as well as the cellular location of the receptor (Doolen et al. 1998; Slominski et al. 2012). However, most of the published studies investigated melatonin receptor mRNA expression solely in total vessel tissue. Distribution and function of MT_1_ and/or MT_2_ protein in the structural layers of the vessel walls remain to be explored comprehensively, but so far there are only a few attempts at cellular allocation of the melatonin receptors in thoracic aorta of rats (Schepelmann et al. 2011) and in human cerebral vessels (Savaskan et al. 2001). The receptors were detected not only in the tunica media and endothelial layer, but also in the adventitia, suggesting an impact of melatonin on vessels from outside (Schepelmann et al. 2011).

Most vessels are surrounded by perivascular adipose tissue (PVAT), which has traditionally been considered to provide mechanical protection to the vessels during contraction of neighboring tissues (Szasz and Webb 2012). It was shown that disruption of PVAT might be a cause as well as a consequence of various vascular pathologies; for example, in clinical practice, the occurrence of vasospasms is well documented when PVAT is removed during isolation of vessel grafts before their chirurgical use (Loesch and Dashwood 2018). Recent studies show that PVAT releases many molecules which affect the contraction of the vessels (Agabiti-Rosei et al. 2018). For example, adiponectin and other adipocyte-derived relaxing factors open K_(v)_ channels in arteries and thus relax aortic and mesenteric rings (Fésüs et al. 2007). Leptin is another important adipokine which can affect the central nervous system and increase sympathetic activity, BP, and heart rate (Haynes 2000; Mark et al. 1999; Simonds et al. 2014). Recent studies suggested that melatonin affects the anti-contractile function of the mesenteric artery (MA)-associated PVAT (Agabiti-Rosei et al. 2014). While evidence for MT_1_ and MT_2_ mRNA expression in adipocytes isolated from rat inguinal and epididymal fat (Zalatan et al. 2001), as well as MT_1_ function in adipocytes derived from epididymal fat, has been obtained (Alonso-Vale et al. 2005), the expression of melatonin receptors in PVAT has never been investigated before.

Therefore, the aim of our study was to evaluate mRNA and protein expression of MT_1_ and MT_2_ by RT-PCR and Western blot in MA as a model for resistance vessels. In addition to the vessel wall, we evaluated the presence of melatonin receptors also in MA-surrounding PVAT. Based on the observed sole expression of MT_1_ mRNA, we further investigated MT_1_ localisation by immunofluorescence microscopy. Moreover, we explored the effects of melatonin on neurogenic contractions generated in isolated superior MA in the absence and presence of PVAT in vitro.

## Methods

### Animals

Male normotensive Wistar rats were obtained from Anlab Praha (Czech Republic) at the age of 16 weeks. Rats were kept in groups of three or four with food and water provided ad libitum in an animal room with controlled temperature (21 ± 2 °C), relative humidity (55 ± 10%), and light intensity (150 lx) under regular 12 h light and 12 h dark conditions. The experimental protocol was approved by the Ethical Committee for the Care and Use of Laboratory Animals at the Comenius University Bratislava, Slovak Republic, and the State Veterinary Authority of Slovak Republic.

### Tissue Sampling

During the light phase, rats (*n* = 6) were sacrificed by administration of ketamine hydrochloride (250 mg/kg). MA without PVAT and PVAT derived from MA (MA-PVAT) were collected, snap frozen in liquid nitrogen, and stored at − 80 °C until RNA or protein were isolated. Likewise, eye and cerebellum samples were collected and served as positive controls for gene and protein expression. The expression of both, MT_1_ and MT_2_, in these tissues is well established (Huan et al. 2001; Schepelmann et al. 2011). Tissue samples for immunofluorescence microscopy were washed briefly in cold phosphate-buffered saline (PBS), fixed in HOPE® (Hepes-glutamic acid buffer mediated Organic solvent Protection Effect; DCS Innovative Diagnostik-Systeme GmbH, Hamburg, Germany) solution, and embedded in paraffin.

### RNA Isolation and RT-PCR

Total RNA was isolated using PeqGOLD® TriFast™ reagent (Peqlab, Erlangen, Germany) according to the manufacturer’s instructions. It was quantified with a NanoDrop™ 1000 (Nanodrop, Wilmington, USA) spectrophotometer and checked for intact 18S and 28S RNA by agarose gel electrophoresis. Total RNA (2 µg) was subjected to reverse transcription using the High-Capacity cDNA Reverse Transcription Kit with RNase Inhibitor (Applied Biosystems, Carlsbad, CA, USA), according to the manufacturer’s instructions. The RT-PCRs for MT_1_, MT_2_, and β-actin were performed using recombinant Taq-polymerase (Fermentas, St. Leon-Rot, Germany), primers, and reaction conditions as published previously (Schepelmann et al. 2011). Individual PCR reactions were performed with cDNA corresponding to 400 ng (MT_1_, 40 cycles), 400 ng (MT_2_, nested PCR; 40 cycle’s outer reaction/20 cycle’s inner reaction), and 50 ng (β-actin, 25 cycles) RNA, respectively.

Negative controls (water instead of cDNA, PCR-neg.co) were used in all reactions. To ensure that the observed amplicons resulted only from reversely transcribed mRNA, samples with no reversely transcribed RNA were included in the PCR setup as well (-RT). Moreover, water instead of RNA was used in the RT reactions (RT-neg.co). The PCR products were analyzed by gel electrophoresis using 1.5% agarose gels containing GelRed and visualized by UV light.

### Real-Time PCR

Expression levels of MT_1_ gene in MA and MA-associated PVAT were compared using real-time quantitative PCR (RT-qPCR). cDNA samples (100 ng) obtained by reverse transcription were amplified using a TaqMan Gene Expression Assay (ID: Rn01488022_m1) and TaqMan Gene Expression Master Mix (Applied Biosystems, by Thermo Fisher Scientific, USA) on a StepOnePlus Real-time PCR System (Applied Biosystem, CA, USA). The experiments were carried out in technical triplicates for each sample (5–6/group). Relative MT_1_ gene expression in MA without PVAT and MA-derived PVAT was calculated using the 2^−∆∆Ct^ method (Livak and Schmittgen 2001). The expression of the housekeeping gene β-actin (ACTB; TaqMan Gene Expression Assay ID Rn00667869_m1; Applied Biosystems, by Thermo Fisher Scientific, USA) was used to normalize the expression of the MT_1_ gene.

### Protein Isolation

Total protein lysates were prepared using T-PER Tissue Protein Extraction Reagent (Thermo Scientific, Rockford, IL, USA) according to the manufacturer’s instructions. Samples were supplied with Halt Protease Inhibitor Cocktail (Thermo Scientific, Rockford, IL, USA) and were stored at − 80 °C until further processing.

### Western Blotting

Protein concentration in the samples was determined with a BCA Protein Assay Kit (Pierce Biotechnology, Rockford, IL, USA). Proteins (25–75 µg) were precipitated with ice-cold acetone and re-dissolved in reducing 1x SDS-sample buffer. Samples were loaded and separated on 12% reducing SDS-PAGE gels and transferred to a BioTrace™ PVDF Transfer Membrane (Pall Corp, Pensacola, FL, USA). Proteins on membranes were stained with Ponceau S and staining patterns were digitized by scanning. Non-specific binding was blocked by incubating the membranes in PBS containing 0.1% (v/v) Tween 20 and 5% (w/v) dry milk (blotto). MT_1_ was detected by incubating membranes with a rabbit polyclonal anti-MT_1_ antibody (amr-031, Alomone laboratories, Jerusalem, Israel, diluted in blotto 1:200) overnight at 4 °C. This antibody is directed against a peptide corresponding to a region of the third intracellular loop (residues 223–236: (C) RVKPDNKPKLKPQD) of mouse MT_1_ receptor, but can also react with rat and human MT_1_ receptor. It has been used to confirm MT_1_ expression in rat retina (Sheng et al. 2015). To confirm specific reaction with human MT_1_ receptor, two identical blots were prepared. The primary antibody solution was split equally into two tubes. The blocking peptide (immunizing antigen) provided with the primary antibody (Alomone laboratories, Jerusalem, Israel) was added to one of the tubes (2 µg blocking peptide per 1 µg primary antibody). After washing with PBS + 0.1% (v/v) Tween 20, membranes were incubated with an appropriate HRP-conjugated secondary antibody (Santa Cruz Biotechnology Inc., Santa Cruz, CA, USA, diluted in blotto 1:2000) for 1 h at room temperature. Blots were washed with PBS + 0.1% (v/v) Tween 20 and chemiluminescence was detected using SuperSignal West Pico Chemiluminescent Substrate (Thermo Scientific, Rockford, IL, USA) and Hyperfilms ECL (GE Healthcare Europe GmbH, Freiburg, Germany).

### Immunofluorescence Microscopy

Paraffin Sects. (4 μm) were prepared, deparaffinized, and rehydrated according to HOPE® manufacturer’s instructions and antigen retrieval was performed by incubation for 20 min in steaming hot 0.05% citraconic anhydride solution at pH 7.4. For blocking and permeabilization, samples were incubated in 5% (v/v) goat serum (Jackson Immuno Research, Newmarket, UK) in PBS containing 0.05% (w/v) saponin (blocking buffer) for 30 min. The sections were then stained with the primary rabbit anti-MT_1_ antibody (amr-031, Alomone laboratories, Jerusalem, Israel, diluted 1:50 in blocking buffer) overnight at 4 °C. When indicated, the blocking peptide provided with the primary antibody was added to the antibody dilution (2 µg blocking peptide per 1 µg primary antibody). A secondary goat anti-rabbit-IgG antibody conjugated to AlexaFluor 647 (Invitrogen molecular probes, Carlsbad, CA, USA; dilution 1:2,000 in blocking buffer) was applied at room temperature for 1 h. Finally, nuclei were stained with 4′,6-diamidino-2-phenylindole dihydrochloride (DAPI; Roche Diagnostics GmbH, Vienna, Austria; dilution 1 µg/mL) at room temperature for 10 min and the sections were mounted in fluoromount G (Sigma-Aldrich, St. Louis, MO, USA). Samples were analyzed with an AxioImager Z1 wide-field microscope (Zeiss, Oberkochen, Germany) equipped with TissueFAXS hardware and image acquisition and management software (Version 4.2; TissueGnostics GmbH, Vienna, Austria). Using a monochrome camera (Hamamatsu, Shizuoka, Japan), grayscale images of each individual fluorescence channel as well as transmitted light images were acquired. Separate pseudocolours were assigned to individual fluorescence images, which were then merged using TissueFAXS software.

### Measurement of Arterial Reactivity Ex Vivo

For the ex vivo experiments, for measurement of arterial contractions, rats were sacrificed under CO_2_ anesthesia and superior MA (*n* = 9) were excised and cut into rings 3–3.5 mm in length. Paired arterial rings were prepared, one with intact PVAT and the other cleaned of PVAT. PVAT was removed under the microscope using fine scissors, being careful not to damage the adventitial layer. The arterial ring preparations were suspended in organ baths filled with oxygenated (95% O_2_ + 5% CO_2_) modified Krebs solution maintained at 37 °C. The arterial rings were set up for measurement of isometric contractile tension using a force–displacement transducer Sanborn FT 10 (Sanborn, Baltimore, USA). The preparations were equilibrated under a resting tension of 10 mN for 60–90 min, and the solution was changed every 15 min. Neurogenic contractions were induced by electrical field stimulation of periarterial sympathetic nerves. Electrical stimulation was provided by an electronic stimulator ST-3 (Medicor, Hungary) via two platinum electrodes pointed on each side and parallel to the vessel preparation. The following parameters of stimulation were used: square-wave pulses (duration: 0.5 ms), supramaximal voltage (> 40 V), duration of stimulation: 20 s, and frequency of stimulation: 1–32 Hz. The neurogenic (sympatho-adrenergic) origin of the contractile responses was confirmed pharmacologically, as described in Török and Zemančíková (2016), indicating that contractile responses were elicited mainly by endogenous norepinephrine released from electrically stimulated periarterial adrenergic nerves. The frequency–response curve was constructed in superior MA preparations with both PVAT intact and removed, first in control conditions, and subsequently after 20 min incubation with melatonin (0.1 mmol/l).

### Statistical Analysis

Data distribution of the RT-qPCR experiment is shown by box plots. Results of the ex vivo experiments are expressed as mean ± SEM. Arterial contractile responses were expressed as active wall tension in mN and normalized to the length of the particular arterial preparation (in mm). The area under the curve (AUC, in arbitrary units) was calculated from individual frequency–response curves in each experimental group using the rectangular rule for numerical integration.

Statistical analysis was performed by one-way analysis of variance (ANOVA) or by paired sample *t* test (responses before and after incubation with melatonin). The differences were considered significant at *p* < 0.05.

## Results

### Expression of Melatonin Receptor MT_1_, But Not MT_2_, mRNA in MA and MA-Associated PVAT

**Fig. 1:**
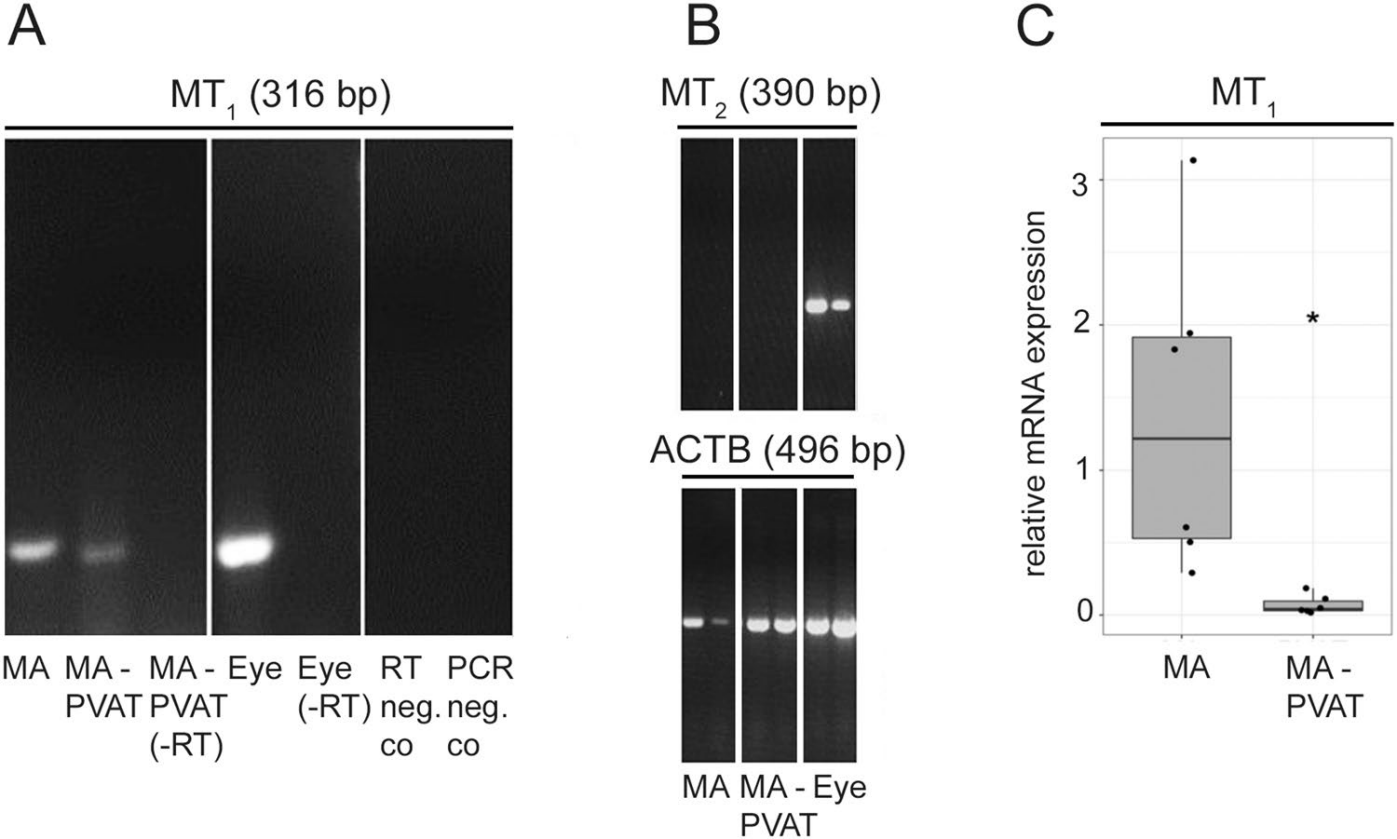
The MT_1_, but not MT_2_, gene is expressed in mesenteric artery (MA) and perivascular adipose tissue (PVAT) derived from MA (MA-PVAT). **a** RT-PCR analysis of MT_1_ mRNA expression in MA, MA-PVAT, and eye. Total RNA isolated from rat tissues was subjected to RT-PCR using specific primers for MT1 (316 bp) cDNA. Non-reversely transcribed samples (-RT, RNA instead of cDNA) and negative controls (PCR-neg.co, water instead of cDNA; RT-neg.co, water instead of RNA) are also shown. **b** RT-PCR analysis of MT_2_ mRNA expression in MA, PVAT derived from MA (MA-PVAT), and eye. Total RNA isolated from rat tissues was subjected to RT-PCR using specific primers for MT_2_ (390 bp; nested PCR, upper panel) and β-actin (496 bp; ACTB, lower panel) cDNAs. Two individual samples per tissue are shown. **c** Relative gene expression levels of MT_1_ in rat MA (*n* = 6) and PVAT derived from MA (MA-PVAT, *n* = 5). The experiments were carried out in technical triplicates for each sample. The distribution of data is displayed by box plots. The box represents the range from the first to third quartiles; the band near the middle of the box is the median, and the lines above and below the box indicate the locations of the minimum and maximum value. The asterisk indicates a significant difference (*p* < 0.05)

MA and its associated PVAT (MA-PVAT) were found to express MT_1_ mRNA (Fig. 1a). As a positive control, rat eye was included. In contrast, MT_2_ mRNA expression was absent from MA as well as the associated PVAT (Fig. 1b), while positive amplification of MT_2_ cDNA from total eye RNA under the same condition could be demonstrated. Positive amplification of β-actin (ACTB) cDNA indicated correct processing of these RNA samples (Fig. 1b). All PCRs were repeated at least 3 times. We observed significantly lower (*p* = 0.034) gene expression of MT_1_ in MA-PVAT compared to MA with PVAT removed (Fig. 1c).

### Expression of MT_1_ Protein in MA and MA-Associated PVAT

**Fig. 2:**
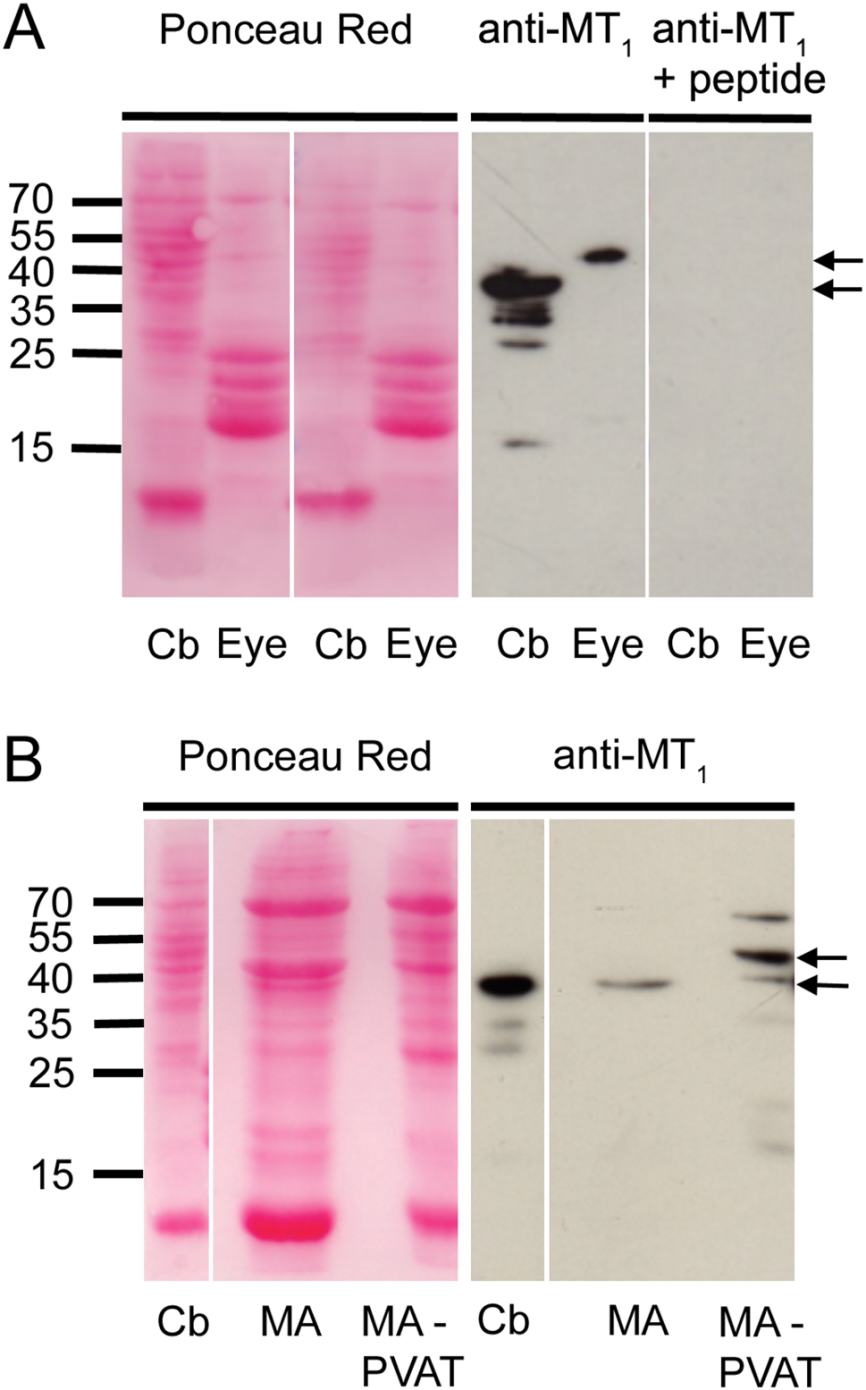
MA and MA-PVAT express MT_1_. **a** Rat cerebellum (Cb) and eye were used to validate the antibody. Total tissue lysates (25 µg/lane) were probed with anti-MT_1_ antibody (left panel) or anti-MT_1_ antibody in the presence of the specific blocking peptide (right panel) by Western blot. **b** Total tissue lysates of rat Cb (20 µg/lane), MA (75 µg/lane), and MA-PVAT (75 µg/lane) were probed with anti-MT_1_ antibody. Arrows point at the two major bands of approximately 40 kDa and 50 kDa, respectively, detected in the tissues

The specificity of the anti-MT_1_ antibody for human MT_1_ was assessed in lysates of human eye and cerebellum. The detected bands were abolished after the antibody was pre-absorbed with the immunogen peptide (Fig. 2a). Expression of MT_1_ receptor protein was shown in MA and MA-associated PVAT (Fig. 2b).

### Melatonin Receptor MT_1_ Localization in Mesenteric Arteries

**Fig. 3:**
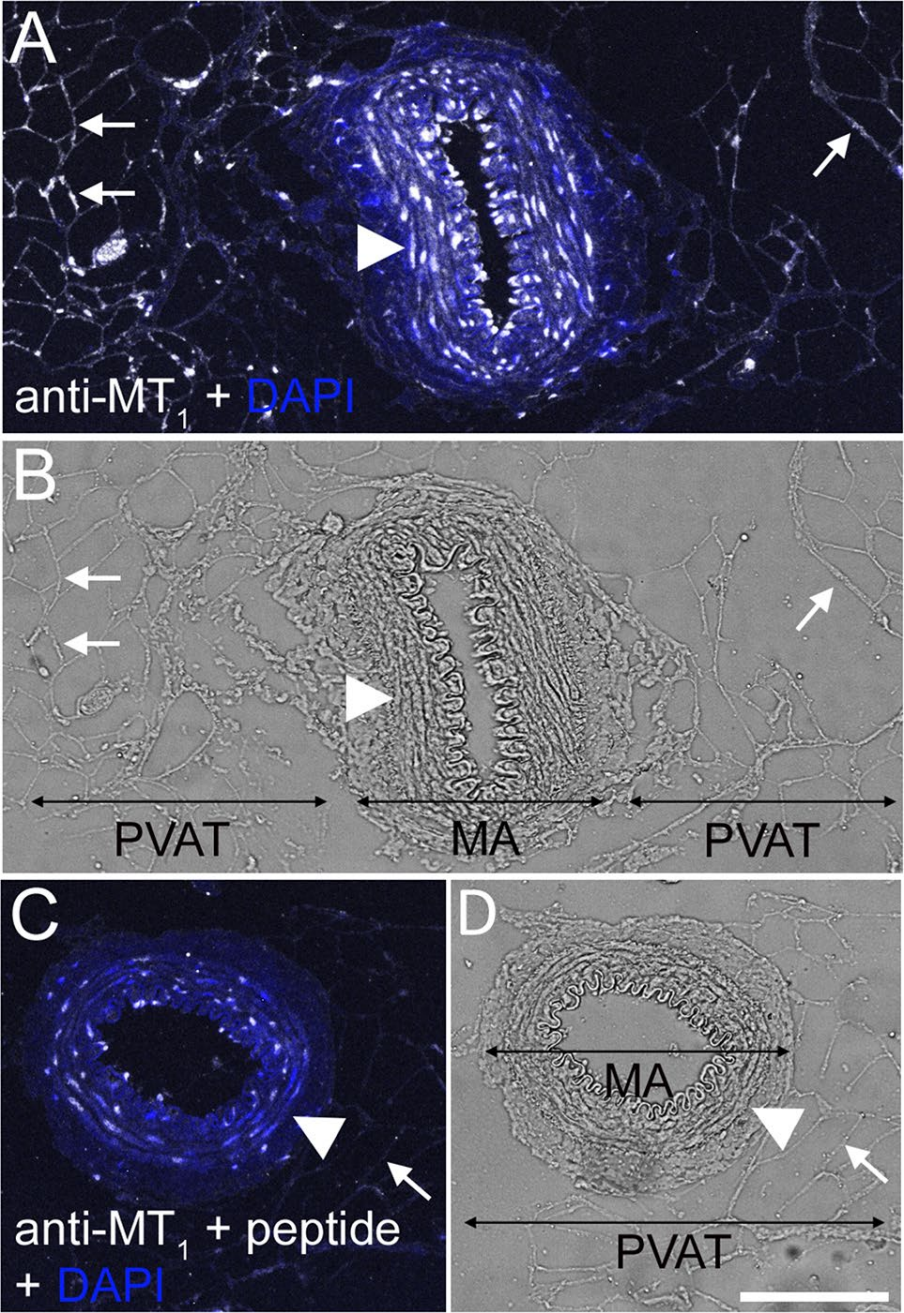
MT_1_ protein localization in smooth muscle layer of rat MA and associated PVAT. Tissue Sects. (4 μm) were stained with anti-MT_1_ antibody and a corresponding AlexaFluor647-conjugated secondary antibody (white staining). Nuclei were stained with DAPI (blue staining). **a** MA and associated PVAT stained for MT_1_. **b** Transmission light micrograph corresponding to (**a**). **c** Significantly reduced MT_1_ staining in MA and associated PVAT after pre-incubation of the anti-MT_1_ antibody with the corresponding blocking peptide. **d** Transmission light micrograph corresponding to (**c**). Arrows indicate adipocytes (examples), and arrowheads point to smooth muscle cells. Bar is 100 µm

MT_1_ receptor immunoreactivity was detected in MA-associated PVAT, but also gave a strong signal in the smooth muscle layer of the MA (Fig. 3a, b). Staining with MT_1_ antibodies was strongly reduced by pre-absorbing the primary antibody with the immunogen peptide (Fig. 3c, d).

### Effect of Melatonin on Neurogenic Contractions in Isolated Superior MA

**Fig. 4:**
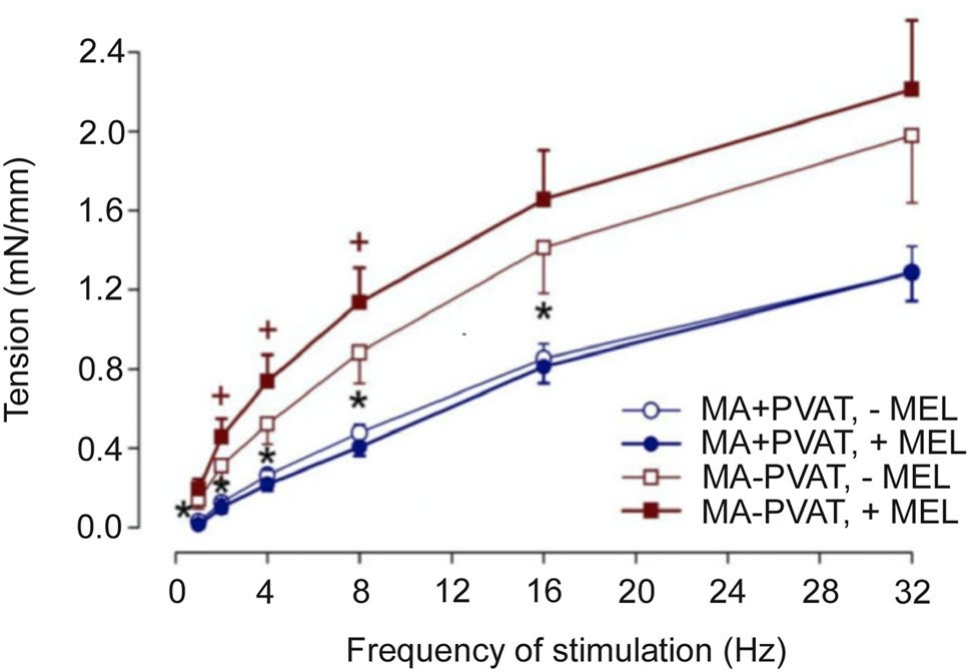
Frequency–response curves to transmural electrical stimulation in rat superior MA with intact PVAT (+ PVAT) and removed PVAT (- PVAT), before and after their incubation with melatonin (MEL). Values represent the mean ± SEM of 9 rats. *p < 0.05 arterial preparations with PVAT intact vs. PVAT removed (before melatonin incubation); +*p* < 0.05 before vs. after melatonin incubation (arteries without PVAT)

The frequency-dependent contractile responses to electrical field stimulation were significantly smaller in mesenteric arterial preparations with intact PVAT (AUC 2.38 ± 0.22) in comparison to those with removed PVAT (AUC 4.19 ± 0.74) (*p* < 0.05; Fig. 4).

In arterial preparations without PVAT, acute melatonin pre-incubation elicited an increase in contractile responses to electrical field stimulation, while in arterial preparations with intact PVAT, melatonin had no potentiating effect on contractile responses (Fig. 4).

## Discussion

### Melatonin Receptor MT_1_, But Not MT_2_, is Present in MA

Melatonin can influence the cardiovascular system via different pathways including MT_1_ and MT_2_ receptors in vessels (Slominski et al. 2012). The general presence of both receptors in vessels and opposite effects of MT_1_ (vasoconstriction) and MT_2_ (vasodilatation) in BP regulation in vitro (Doolen et al. 1998) as well as in vivo (Masana et al. 2002) has been expected.

In our experiments, we evaluated expression of both receptors in MA, which belong to the resistant part of the cardiovascular tree and substantially contributes to BP control (Intengan and Schiffrin 2001).

Our results revealed the presence of MT_1_ but not MT_2_ mRNA and protein in MA. These data are in line with our previous results, where we have shown the absence of MT_2_ in the conduit thoracic artery (Schepelmann et al. 2011). The distribution pattern of MT_1_ however, seems to be different between conduit and resistance arteries. In the conduit thoracic artery, MT_1_ is present predominantly in the adventitia and in the endothelial cell layer (Schepelmann et al. 2011), while we observed a strong signal for MT_1_ in the smooth muscle layer of MA.

Rat MT_1_ is composed of 353 amino acids (Audinot et al. 2008; Reppert et al. 1994), which results in a proposed molecular weight (MW) of 40 kDa for the unglycosylated rat MT_1_ protein. Indeed, a MW of 37–40 kDa has been demonstrated by Western blotting for MT_1_ in several studies (Hill et al. 2013; Fujieda et al. 1999; Richter et al. 2008; Sanchez-Hidalgo et al. 2009). As MT_1_ contains two consensus sites for N-terminal asparagine-linked glycosylation (Ishii et al. 2009), proteins with higher MW (40–60 kDa) might be expected as well. For human MT_1_, a MW of 60 kDa has been shown by Western blotting (Brydon et al. 1999). In our study, total protein lysates of eye and cerebellum were used as MT_1_-positive tissue samples (Huan et al. 2001; Schepelmann et al. 2011). While a band of approximately 50 kDa was detected in eye, a strong band of approximately 40 kDa as well as some bands of lower molecular weight were detected in cerebellum. In analogy to the study of Sheng et al. (Sheng et al. 2015), pre-incubation of the primary antibody with the blocking peptide totally abolished binding of the antibody, confirming specificity of the antibody for MT_1_ protein.

MA and MA-PVAT also gave positive signals with the anti-MT_1_ antibody. A band of approximately 40 kDa was detected in MA and MA-PVAT lysates, while in the MA-PVAT sample additional bands were found, the strongest band corresponding to a MW of approximately 50 kDa. The localization of MT_1_ protein in MA and MA-associated PVAT was also confirmed by immunofluorescence microscopy, where we found expression in the smooth muscle cells and the adipocytes. Use of the blocking peptide significantly reduced binding of the antibody, confirming specificity of antibody binding.

The presence of MT_1_ receptors in the superior MA prompted us to explore melatonin effects under ex vivo conditions in relation to the surrounding PVAT. Acute administration of melatonin had no effect on the basal tone (data not shown); however, it modulated neurogenic contractile responses. In arterial preparations cleaned of PVAT, a significant increase in these contractions was observed after pre-treatment with melatonin. These results are in accordance with our findings of MT_1_ receptor expression in vascular smooth muscle cells where the receptor can mediate melatonin-induced pro-contractile effects. In arterial preparations with intact PVAT melatonin had no potentiating effect on contractile responses, suggesting an anti-contractile effect of surrounding PVAT.

Vasoactive effects of melatonin may depend on the type of vessel. Based on studies performed on caudal arteries, activation of MT_1_ receptors was suggested to induce vasoconstriction, while, in contrast, vasodilation was suggested to occur as a consequence of MT_2_ activation (Doolen et al. 1998; Masana et al. 2002). Moreover, the effects of melatonin can be dose dependent, since lower doses of melatonin activated MT_2_ receptors, while higher melatonin concentrations stimulated also MT_1_ receptors in the rat pial microvascular network (Lapi et al. 2011). Therefore, the effects of melatonin on blood vessels might be dose dependent and vessel-type specific.

### The Role of PVAT

A variable action of melatonin in different types of vessels can be explained by unequal distribution of melatonin receptors in their wall and by melatonin action on PVAT surrounding these vessels. In contrast to the previously assumed sole role of mechanical support for the blood vessels, recent studies demonstrated that PVAT can regulate vascular smooth muscle tone through the release of adipocyte-derived relaxing and contracting factors, which are not well described (Brown et al. 2014). In humans, PVAT can have vasorelaxant effects and therefore its impaired anti-contractile action can be involved in the pathogenesis of hypertension (Oriowo 2015).

PVAT surrounding the MA consists of white adipose tissue. The major functions of this depot are (1) lipid storage by utilization of circulating free fatty acids, triglycerides, and glucose; (2) “buffering” of inflammation induced by macrophage infiltration; and (3) autocrine/paracrine secretion of adipokines. Adipokines secreted from adipocytes may impact on vessel function by acting on immune cells, stem cells and fibroblasts in the adventitia, smooth muscle cells in the media and, consequently, implicate PVAT in BP control (Brown et al. 2014; Oriowo 2015; Szasz and Webb 2012).

Melatonin thus could influence the anti-contractile function of MA-associated PVAT via melatonin receptors (Agabiti-Rosei et al. 2014, 2017). To the best of our knowledge, this is the first study demonstrating the expression of MT_1_ in MA-associated PVAT. However, the expression of MT_1_ and MT_2_ mRNA was previously demonstrated in adipocytes isolated from the rat inguinal and epididymal fat (Zalatan et al. 2001).

Our ex vivo data showed that in arterial segments with intact PVAT, melatonin had no potentiating effect on neurogenic contractions. An anti-contractile effect of PVAT has been demonstrated also in MA of mice. This was not detected in obese (Agabiti-Rosei et al. 2014) and old (Agabiti-Rosei et al. 2017) individuals, but could be restored after long-term melatonin administration (Agabiti-Rosei et al. 2014, 2017). We hypothesize that melatonin via MT_1_ receptors in PVAT could stimulate the release of PVAT-derived relaxing factors, which can counteract the direct pro-contractile effect of melatonin on vascular smooth muscle. Alternatively, PVAT-dependent anti-contractile action of melatonin may occur via modulation of biosynthesis and release of other vasoactive compounds.

For example, melatonin was shown to upregulate insulin-stimulated leptin synthesis and secretion by interaction with insulin (Alonso-Vale et al. 2005). These effects were mediated via MT_1_ receptors and by co-activation of insulin receptors and the convergent protein Akt by melatonin (Alonso-Vale et al. 2005). However, while leptin is also produced by PVAT (Dashwood et al. 2011), the anti-contractile effect of PVAT was maintained in the Zucker fa/fa rats that lack functional leptin receptors (Löhn et al. 2002).

PVAT also secretes adiponectin. Previously, melatonin was shown to increase the expression of adiponectin and decrease the expression of IL-6 and IL-17 in colonic tissue of mice (Kim et al. 2016). Moreover, adiponectin-deficient mice exhibited an increase in BP (Ohashi et al. 2006). Lynch et al. (Lynch et al. 2013) later demonstrated a mechanistic link between the anti-contractile effect of PVAT and BK_CA_ channels and suggested adiponectin as the relaxing factor. On the other hand, in the study of Fesüs et al. (Fésüs et al. 2007) aortae and mesenteric arteries from adiponectin‐knockout mice have been shown to maintain their anti-contractile properties. It thus remains controversial whether adiponectin is an adipocyte-derived relaxing factor (ADRF).

Overall, PVAT adipocytes release many bioactive signaling molecules with vasoregulatory effect. Simple molecules such as NO, H_2_S, reactive oxygen species [e.g., hydrogen peroxide (H_2_O_2_)], as well as adipokines [e.g., adiponectin, leptin, angiotensin 1–7 (Ang1-7)] may contribute to the anti-contractile effect of PVAT on the vascular bed. However, the exact nature of the ADRF is still not known as concluded by several recent reviews (e.g., Cheng et al. 2018; Agabiti-Rosei et al. 2018; Zaborska et al. 2017). It might even be a combination of several different molecules, depending on the stimulus applied, the vascular bed examined, and the phenotypic state of the PVAT (Xia and Li 2017). Thus, in the future, a thorough and systematic approach is required to identify the most important ADRFs with a mechanistic link to melatonin and MT_1_.

Recently we have shown that in the isolated superior MA, the presence of PVAT caused a reduction in contractions induced by exogenous as well as endogenous norepinephrine, while PVAT exerted no inhibitory effect on these responses in the abdominal aorta (Torok and Zemancikova 2016; Zemancikova and Torok 2019). These differences can partially be related to the different distribution of the sympathetic innervation within the arterial wall and in PVAT (Torok et al. 2016). Therefore, we assume that PVAT can act differently on different types of arteries. PVAT may have a more prominent effect on BP regulation in resistance arteries, such as MA, while in conduit arteries PVAT may rather protect against stiffness.

## Conclusions

Melatonin MT_1_ but not MT_2_ receptor mRNA was found in the MA of mature rats and MT_1_ was localized mainly in smooth muscle cells of the vessel wall. Moreover, we detected MT_1_ receptors also in the PVAT of MA. The presence of MT_1_ receptors corresponds with the expected functions of this pleiotropic compound in the cardiovascular system. Melatonin had no effect on the basal tone of the MA, but mediated pro-contractile responses in arteries devoid of PVAT. In arteries with intact PVAT, melatonin did not affect neurogenic contractions. Melatonin is involved in the control of vascular tone in a complex way, which is vessel specific and can reflect a sum of actions on different layers of the vessel wall and surrounding PVAT.
